# Insights in the Antimicrobial Potential of the Natural Nisin Variant Nisin H

**DOI:** 10.3389/fmicb.2020.573614

**Published:** 2020-10-20

**Authors:** Jens Reiners, Marcel Lagedroste, Julia Gottstein, Emmanuel T. Adeniyi, Rainer Kalscheuer, Gereon Poschmann, Kai Stühler, Sander H. J. Smits, Lutz Schmitt

**Affiliations:** ^1^Institute of Biochemistry, Heinrich-Heine-University Düsseldorf, Düsseldorf, Germany; ^2^Center for Structural Studies, Heinrich-Heine-University Düsseldorf, Düsseldorf, Germany; ^3^Institute of Pharmaceutical Biology and Biotechnology, Heinrich-Heine-University Düsseldorf, Düsseldorf, Germany; ^4^Institute for Molecular Medicine, Medical Faculty, Heinrich-Heine-University Düsseldorf, Düsseldorf, Germany; ^5^Molecular Proteomics Laboratory, BMFZ, Heinrich-Heine-University-Düsseldorf, Düsseldorf, Germany

**Keywords:** lantibiotics, nisin, nisin H, MS analysis, antimicrobial activity

## Abstract

Lantibiotics are a growing class of antimicrobial peptides, which possess antimicrobial activity against mainly Gram-positive bacteria including the highly resistant strains such as methicillin-resistant *Staphylococcus aureus* or vancomycin-resistant enterococci. In the last decades numerous lantibiotics were discovered in natural habitats or designed with bioengineering tools. In this study, we present an insight in the antimicrobial potential of the natural occurring lantibiotic nisin H from *Streptococcus hyointestinalis* as well as the variant nisin H F_1_I. We determined the yield of the heterologously expressed peptide and quantified the cleavage efficiency employing the nisin protease NisP. Furthermore, we analyzed the effect on the modification via mass spectrometry analysis. With standardized growth inhibition assays we benchmarked the activity of pure nisin H and the variant nisin H F_1_I, and their influence on the activity of the nisin immunity proteins NisI and NisFEG from *Lactococcus lactis* and the nisin resistance proteins *Sa*NSR and *Sa*NsrFP from *Streptococcus agalactiae* COH1. We further checked the antibacterial activity against clinical isolates of *Staphylococcus aureus*, *Enterococcus faecium* and *Enterococcus faecalis* via microdilution method. In summary, nisin H and the nisin H F_1_I variant possessed better antimicrobial potency than the natural nisin A.

## Introduction

Lantibiotics (lanthionine containing antibiotics) are a growing class of antimicrobial peptides (AMPs), which posses antimicrobial activity even against highly resistant strains such as methicillin-resistant *Staphylococcus aureus* (MRSA) or vancomycin-resistant *enterococci* (VRE) and some are already in pre-clinical trials (Mota-Meira et al., 2000; Jabes et al., 2011; Dawson and Scott, 2012; Crowther et al., 2013; Dischinger et al., 2014; Ongey et al., 2017; Brunati et al., 2018; Sandiford, 2019). Lantibiotics are peptides, containing 19–38 amino acids and are mainly produced by Gram-positive bacteria (Klaenhammer, 1993; Sahl and Bierbaum, 1998). In the last decades an increasing number of lantibiotic gene clusters were found by data-mining approaches using tools such as BAGEL4 (van Heel et al., 2018).

The best studied lantibiotic is nisin, which was first discovered in 1928 by Rogers and Whittier and belongs to the class I lantibiotics (Rogers, 1928; Rogers and Whittier, 1928; Arnison et al., 2013). It is used in the food industry since 1953 and obtained the status as generally recognized as safe (GRAS) in 1988 from the Food and Drug Administration (FDA) (Delves-Broughton et al., 1996). The class I lantibiotic nisin contains 34 amino acids and five (methyl)-lanthionine rings. These (methyl)-lanthionine rings require multiple posttranslational modifications (PTMs) which are introduced in the precursor peptide. First, the serine and threonine residues in the core peptide are dehydrated by a specific dehydratase NisB (lantibiotic class I LanB dehydratase) (Kaletta and Entian, 1989; Karakas Sen et al., 1999; Koponen et al., 2002; Ortega et al., 2015; Repka et al., 2017). The next step is a Michael-type condensation of dehydrated residues with the thiol group of a cysteine residue, thereby forming (methyl)-lanthionine rings, guided in a regio- and stereospecific manner by the cyclase NisC (class I lantibiotic cyclase) (Okeley et al., 2003; Li et al., 2006; Li and van der Donk, 2007; Repka et al., 2017). These characteristic (methyl)-lanthionine rings give lantibiotics high heat stability, resistance against proteolytic digestion and are responsible for the nanomolar antimicrobial activity (Gross and Morell, 1967; Rollema et al., 1995; Chan et al., 1996; Lu et al., 2010; Oppedijk et al., 2016).

**FIGURE 1:**
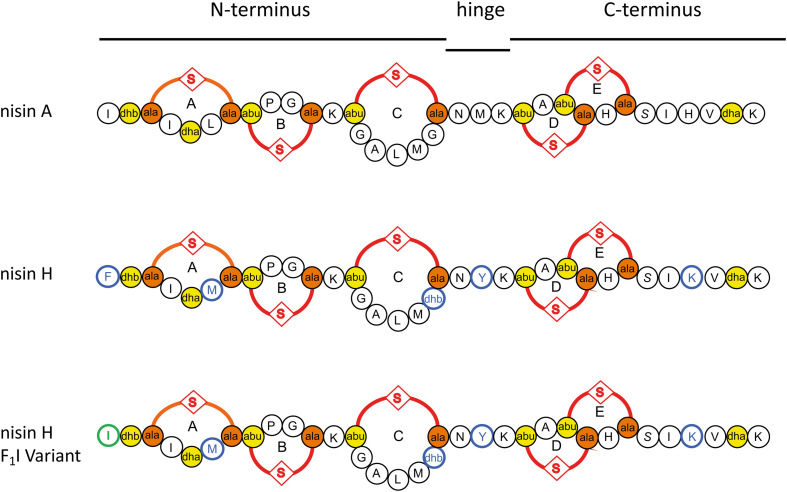
Schematic overview of the used lantibiotics nisin A, nisin H, and the nisin H F_1_I variant. Point mutations in nisin H and nisin H F_1_I in comparison to nisin A are highlighted in blue. Dehydrated amino acids and cysteine residues involved in (methyl)-lanthionine ring formation (ring A, B, C, D, and E) are labeled in yellow and orange. The mutation of the nisin H F_1_I variant is highlighted in green.

The sequence of nisin A can be subdivided into three parts (see Figure 1). The N-terminal part with ring A, B, and C is responsible for binding to the cell wall precursor lipid II (Hsu et al., 2004). The hinge region is very flexible and allows reorientation of the C-terminal part to insert into the membrane (van Heusden et al., 2002; Hasper et al., 2004; Wiedemann et al., 2004; Medeiros-Silva et al., 2018), while changes in this region have a strong impact on the target antimicrobial activity (Zhou et al., 2015; Zaschke-Kriesche et al., 2019b). After penetrating the membrane, the C-terminal part with ring D and E forms a stable pore with a stoichiometry of eight nisin and four lipid II molecules, which subsequently leads to rapid cell death (Hasper et al., 2004; Wiedemann et al., 2004; Alkhatib et al., 2014a; Medeiros-Silva et al., 2018).

Unfortunately, some bacteria established resistance mechanism against lantibiotics. For instance lantibiotic producing strains express the immunity system LanI and LanFEG (Alkhatib et al., 2014a,b), which prevent a suicidal effect after the lantibiotic is secreted. In the case of nisin A from *Lactococcus lactis* these proteins are called NisI and NisFEG. But also non-lantibiotic producing strains showed resistance against lantibiotics like *Streptococcus agalactiae* COH1, which arises from the expression of the membrane-anchored peptidase *Sa*NSR and the ABC transporter *Sa*NsrFP (Khosa et al., 2013, 2016a,b; Reiners et al., 2017).

Several natural nisin variants have been discovered and up to now eight are known. First of all nisin A from *L. lactis* (Rogers and Whittier, 1928), nisin Z from *L. lactis* NIZO 221 86 (Mulders et al., 1991), nisin F from *L. lactis* F10 (de Kwaadsteniet et al., 2008), nisin Q from *L. lactis* 61-14 (Zendo et al., 2003), nisin O_1_ to O_4_ from *Blautia obeum* A2-162 (Hatziioanou et al., 2017), nisin U and U2 from *Streptococcus uberis* 42 and D536 (Wirawan et al., 2006), nisin P from *Streptococcus gallolyticus* subsp. *pasteurianus* (Zhang et al., 2012; Wu et al., 2014), nisin J from *Staphylococcus capitis* APC 2923 (O’Sullivan et al., 2020) and nisin H from *Streptococcus hyointestinalis* DPC 6484 (O’Connor et al., 2015).

In this study we focused on the natural nisin H variant (Figure 1). We used a standardized workflow for the characterization of lantibiotics, previous described in Lagedroste et al. (2019) to determine the impact on the expression, modification and antimicrobial properties of this nisin variant. We tested further the antimicrobial activity against some pathogen strains from *Staphylococcus aureus*, *Enterococcus faecium*, and *Enterococcus faecalis* using the microdilution method. As a reference we used the wild-type version of nisin A expressed using the same experimental setup. Furthermore, we exchanged the phenylalanine at position one (F_1_) of nisin H to isoleucine, which is the natural amino acid of nisin A at this position (Figure 1). This position one was previously analyzed in nisin A and showed a major impact on different levels of the characterization (Lagedroste et al., 2019).

## Materials and Methods

### Microorganisms and Culture Conditions

Cultures of *L. lactis* NZ9000 (Kuipers et al., 1997) containing the plasmids for immunity and resistance proteins were grown in M17 medium (Terzaghi and Sandine, 1975) at 30°C supplemented with 0.5% glucose [GM17 and the appropriate antibiotics descripted in Alkhatib et al. (2014a, b), Khosa et al. (2016b), Reiners et al. (2017), Lagedroste et al. (2019)]. For pre-nisin secretion, the *L. lactis* strain NZ9000 was grown in minimal medium (Jensen and Hammer, 1993) at 30°C supplemented with 0.5% glucose and the appropriate antibiotics. All bacteria used for minimum inhibitory concentration (MIC) determination of nisin variants [*Bacillus subtilis* 168; *S. aureus*: MSSA strain ATCC 29213, MRSA/VISA strain ATCC 700699; *E. faecium*: ATCC 35667, ATCC 700221 (vancomycin resistant); *E. faecalis*: ATCC 29212, ATCC 51299 (vancomycin resistant)] were cultivated in Mueller-Hinton broth (MHB) at 37°C and shaking at 150 rpm.

### Cloning of Nisin H and the F_1_I Variant

Nisin H was created as described in Reiners et al. (2017). The substitution of the phenylalanine at position one (F_1_I) to an isoleucine was performed by site-directed mutagenesis. Here, we used the following primers (forward: 5′-GTGCATCACCACGCTTTACAAGTATTTCGC-3′; reverse: 5′-GCGAAATACTTGTAAAGCGTGGTGATGCAC-3′). After sequence analysis a competent *L. lactis* NZ9000 strain was transformed with the resulting plasmid via electroporation (Holo and Nes, 1989). The *L. lactis* NZ9000 strain already contain a vector (pil3-BTC) encoding for the modification and secretion proteins (Rink et al., 2005).

### Expression, Purification and Activation of Pre-nisin Variants

The precursor of nisin H and its variant were expressed and purified as previously described (Abts et al., 2013; Alkhatib et al., 2014b; Lagedroste et al., 2017, 2019). Briefly, for pre-nisin secretion, the *L. lactis* strain NZ9000 was grown in minimal medium (Jensen and Hammer, 1993) supplemented with 0.5% glucose and 5 μg/ml of each erythromycin and chloramphenicol at 30°C. Cells were induced with 10 ng/ml nisin at an OD_600_ of 0.4 and further grown overnight at 30°C without shaking. After harvesting the cells, the 0.45 μm-filtered supernatant was loaded onto a HiTrap SP HP cation exchange chromatography column. After washing with 50 mM lactic acid, the buffer was changed to 50 mM Hepes pH 7 via gradient and the final elution was done with 50 mM Hepes pH 7, 500 mM NaCl buffer. Elution fractions were concentrated in a 3 kDa cutoff filter. With a soluble version of NisP, the activation of all variants was performed overnight at 8°C (Lagedroste et al., 2017). The yield and cleavage efficiency determination was done by RP-HPLC (Agilent Technologies 1260 Infinity II) with a LiChrospher WP 300 RP-18 end-capped column and an acetonitrile/water solvent system (Abts et al., 2013; Lagedroste et al., 2017, 2019).

### MALDI-TOF Analysis: Dehydration and (Methyl)-Lanthionine Ring Analysis

With MALDI-TOF analysis we analyzed the modification state of nisin H and its variant. Dehydrations are directly visible in the spectra, due to the loss of mass (−18 Da). For the determination of the presence of (methyl)-lanthionine rings, we used the organic coupling agent CDAP (1-cyano-4 dimethylaminopyridinium tetrafluoroborate) that binds to free cysteine residues (Wu and Watson, 1998). The reaction of the coupling agent to free cysteine side chains would results in an increased mass in the spectra. The analysis was performed as previously descripted (Lagedroste et al., 2019). The samples were analyzed with MALDI-TOF (UltrafleXtreme, Bruker Daltonics, Bremen, Software: Compass 1.4) in positive mode.

### Tandem Mass Spectrometric Analysis of Nisin H and Nisin H F_1_I

Nisin H and the nisin H F_1_I variant were purified using solid phase extraction (Oasis HLB, Waters) and finally resuspended in 0.1% trifluoroacetic acid. The samples were first subjected to liquid chromatography on a rapid separation liquid chromatography system (Ultimate 3000, Thermo Fisher Scientific) using an 1 h gradient and C18 columns as described (PMID 24646099) and further analyzed by a QExactive Plus mass spectrometer (Thermo Fisher Scientific) coupled via a nano-source electrospray interface. First, a precursor spectrum was acquired at a resolution of 140,000 (advanced gain control target 3E6, maximum ion time 50 ms, scan range 200–2000 m/z, profile data type). Subsequently, up to four 4–6-fold charged precursors were selected by the quadrupole (2 m/z isolation window), fragmented by higher-energy collisional dissociation (normalized collision energy 30) and recorded at a resolution of 35,000 (advanced gain control target 1E5, maximum ion time 50 ms, available scan range 200–2000 m/z, centroid data type).

Recorded spectra were analyzed by the MASCOT search engine (version 2.4.1, Matrix Science) and searches triggered by Proteome Discoverer (version 2.4.0.305, Thermo Fisher Scientific). A database was generated for the searches including 1000 randomly generated sequence entries each 34 amino acid long) and the sequences of nisin H and nisin H F_1_I. Methionine oxidation and dehydration of serine and threonine residues were considered as variable modifications and the precursor mass tolerance set to 10 ppm and the fragment mass tolerance set to 0.02 Da. For peptide validation, the Fixed Value PSM validator was used (1% false discovery rate) and the IMP-ptmRS node for site validation (PMID 22073976). No random sequences were found by the search.

### Determination of the Antimicrobial Activity by Growth Inhibition Assay

The determination of the antimicrobial activity of the different nisin variants was tested using a growth inhibition assay. The used strains were described in Alkhatib et al. (2014a, b), Reiners et al. (2017), and Lagedroste et al. (2019).

Briefly, the *L. lactis* NZ9000 strains were grown in M17 medium (Terzaghi and Sandine, 1975) at 30°C supplemented with 0.5% glucose (GM17 and the appropriate antibiotics) overnight with 1 ng/ml nisin. In a 96-well plate, a serial dilution of the nisin variant was applied and incubated with the test strains at a final OD_600_ of 0.1 for 5 h at 30°C. Later on, the optical density was measured at 584 nm via 96-well plate reader BMG. The normalized optical density was plotted against the logarithm of the nisin concentration and the resulting inhibitory concentration (IC_50_), represents the value where 50% of the cells died in the presence of the different nisin variants. By dividing the IC_50_ values of strains expressing the immunity or resistance proteins from the IC_50_ value of the sensitive strain we calculated the fold of immunity/resistance, which is an indicator for the recognition of nisin H or its variant by the immunity or resistance proteins.

### Minimum Inhibitory Concentration Determination of Nisin Variants

Nisin variants were tested for antibacterial capabilities against *B. subtilis* and different strains from *S. aureus*, *E. faecium*, and *E. faecalis* using the microdilution method, according to the recommendations of Clinical and Laboratory Standards Institute (2012). Briefly, fresh cultures prepared from overnight cultures were incubated until exponential phase (OD ∼ 0.6) and seeded at 5 × 10^4^ CFU/well in 96-well round-bottom microplates, in a total volume of 100 μL containing twofold serially diluted test peptides. Moxifloxacin was used as a positive control. Plates were incubated statically and aerobically for 24 h at 37°C. MICs were determined macroscopically by identifying the least concentration of peptides that resulted in complete inhibition of bacterial visual growth.

### SYTOX Green Nucleic Acids Binding Assay

The cells of NZ9000Cm were grown overnight in GM17 supplemented with 5 mg/ml chloramphenicol. The overnight culture was diluted to an OD_600_ of 0.1 in fresh media and the cultures were grown until the OD_600_ reaches 0.3. The SYTOX green dye was added at a final concentration of 2.5 mM according to the manual of the manufacturer (Invitrogen). After reaching a stable baseline (∼200 s) we added 100 nM of the nisin variants. The fluorescence signal was measured at an excitation wavelength of 504 nm and emission wavelength of 523 nm, respectively (using a fluorolog Horiba III). After a stable baseline was reached, the nisin variant was added and the fluorescence was monitored over an additional time period. The measurement was performed at 30°C.

## Results

O’Connor et al. (2015) described a new natural nisin variant from *S. hyointestinalis* DPC 6484 and named it nisin H. In following, we compared nisin A and its natural variant nisin H, both heterologously produced in *L. lactis*, following the protocol of lantibiotic characterization (Lagedroste et al., 2019). We also included the nisin H F_1_I variant.

**FIGURE 2:**
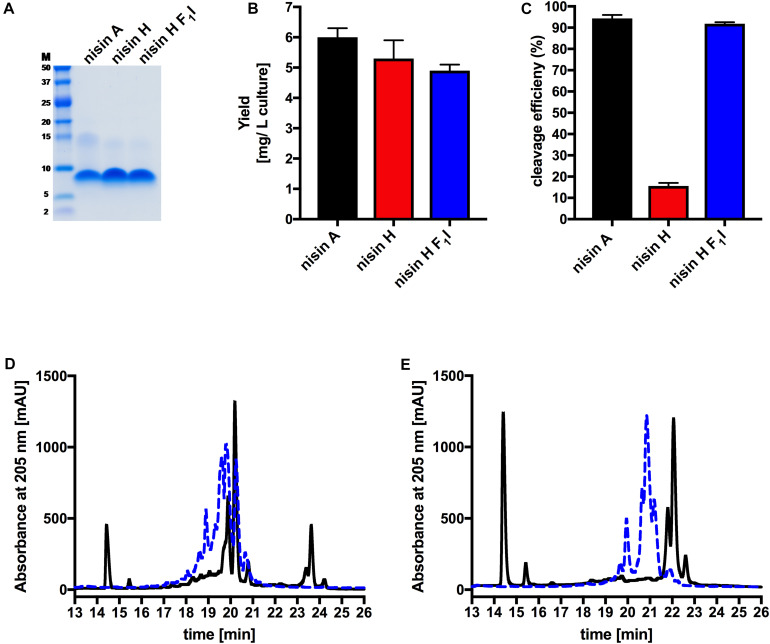
Determination of purity, yield and cleavage efficiency of the pre-nisin variants. **(A)** Purity of the purified nisin A and the variants nisin H and nisin H F_1_I (Marker: Precision Plus Protein Dual Xtra standards Bio-Rad). **(B)** Yields after purification of nisin A and their corresponding variants via cation-exchange chromatography. **(C)** Quantification of the cleavage efficiency of NisP. **(D)** Chromatogram of nisin H. **(E)** Chromatogram of the nisin H F_1_I variant. The pre-nisin variants before NisP cleavage were shown by blue dashed lines and after NisP cleavage by black lines. Error bars represent the standard deviation of at least three biological replicates.

The characterization starts with the expression, secretion and purification of the lantibiotic and its comparison to nisin A. The heterologously expressed and secreted nisin A and the variants nisin H and nisin H F_1_I can be purified with high purity as observed on the Tricine-SDS-PAGE gel (Figure 2A). Nisin A was purified with a yield of 6.0 ± 0.3 mg/L of cell culture (Lagedroste et al., 2019), nisin H was expressed and purified with a yield of 5.3 ± 0.6 mg/L of cell culture, which is identical within experimental error. The nisin H F_1_I variant displayed a slightly reduced yield of 4.9 ± 0.2 mg/L of cell culture (Figure 2B and Table 1).

**TABLE 1:** Determination of the yield, cleavage efficiency, dehydrations, and (methyl)-lanthionine ring formation for nisin A, nisin H, and nisin H F_1_I.

**Variant**	**Yield (mg/L culture)**	**Cleavage (%)**	**Dehydrations**	**Lanthionine rings**
Nisin A	6.0 ± 0.3	94.6 ± 2.0	**8**, 7	5
Nisin H	5.3 ± 0.6	15.6 ± 1.4	9, **8**, 7	5
Nisin H F_1_I	4.9 ± 0.2	91.8 ± 0.8	**9**,8,7,6	5

*Main species found in MALDI-TOF analysis are marked in bold.*

An important step prior to the activity assay is the cleavage of the leader sequence form the pre-nisin variants, resulting in biologically active compounds. For the cleavage reaction, we used the peptidase NisP and monitored the cleavage efficiency via RP-HPLC (Figures 2C–E).

Intriguingly, the natural variant nisin H showed only a low cleavage efficiency of 15.6 ± 1.4%, compared to nisin A with 94.6 ± 2.0% (Figure 2C and Table 1). In comparison to nisin A, nisin H contains a phenylalanine at the first position (O’Connor et al., 2015), which apparently leads to a significant reduction in cleavage efficiency. The first residue of nisin A is an isoleucine, and as demonstrated before (Lagedroste et al., 2019), the introduction of aromatic residues at position one results in a clearly reduced cleavage efficiency. To counteract the lower cleavage efficiency of nisin H by NisP, we created a mutant of nisin H, in which the phenylalanine was substituted by isoleucine and termed it nisin H F_1_I. For this variant, nisin H F_1_I, the cleavage efficiency of the pre-lantibiotic was restored with an efficiency of 91.8 ± 0.8% (Figure 2C and Table 1), which corresponds to levels previously observed for nisin A. We monitored the cleavage via RP-HPLC, the pre-nisin elutes normally between 18 and 22 min (shown as blue dashed lines, Figures 2D,E). After cleavage by NisP, the leader peptide can be detected at 14.5 and 15.5 min in the chromatogram (shown as black lines, Figures 2D,E). For nisin H there was a high amount of uncleaved nisin H visible (eluting from 18 to 21 min) and only a small amount of cleaved product at 23–24 min (black lines, Figure 2D). For the nisin H F_1_I variant, high amounts of leader peptide and cleaved product could be detected in the chromatogram, indicating high cleavage efficiency (black lines, Figure 2E). This efficiency was similar as observed for nisin A and in line with previous results that the position one is important for the cleavage reaction (Lagedroste et al., 2019). Thus, we assume, that the four other mutations naturally occurring in nisin H (compared to nisin A) do not interfere with cleavage, however the isoleucine at position one does.

**FIGURE 3:**
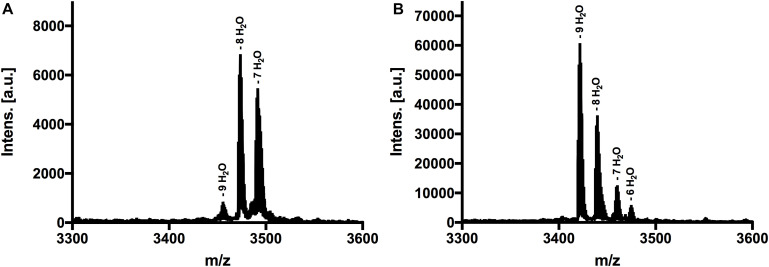
MALDI-TOF analysis to determine dehydrations and (methyl)-lanthionine ring formations. **(A)** MS spectra of nisin H, threated with CDAP. Dominant species was eightfold dehydrated. **(B)** MS spectra of nisin H F_1_I variant, threated with CDAP. Dominant species was ninefold dehydrated. Both variants showed no coupling products, indicating that all cysteine residues were involved in (methyl)-lanthionine rings.

The next step was to determine the modification state of the heterologous produced nisin H and its F_1_I variant. As the natural variant nisin H contains ten possible residues in the core peptide that can be dehydrated, we were curious to determine if the modification machinery of nisin A was able to modify the peptide as efficiently (O’Connor et al., 2015). In the MALDI-TOF spectra, a dominant species of eightfold dehydrated residues was observed for nisin H, followed by a minor species containing nine- and sevenfold dehydrations. The possible 10-fold dehydrated species however was not observed (Figure 3A and Table 1). Furthermore no CDAP-coupling products were observed, which indicates that all cysteine residues are linked in (methyl)-lanthionine rings. We proved the functionality of the assay with unmodified pre-nisin A as demonstrated in Lagedroste et al. (2019). Thus, the modification enzymes were able to modify nisin H proving the promiscuity of the nisin modification machinery. Interestingly, the nisin H F_1_I variant showed a dominant ninefold dehydrated species in comparison the nisin H wild-type (Figure 3B and Table 1) and also showed no CDAP-coupling products, indicating that all cysteine residues are closed to (methyl)-lanthionine rings. The difference in the dehydration pattern for the nisin H F_1_I variant indicates a different accessibility of the serine and/or threonine residues in the core peptide for at least the dehydratase NisB. To validate which serine or threonine residues is dehydrated, we performed a tandem mass spectrometric analysis of nisin H and the F_1_I variant. Here we found that the Thr_2_ partially escape the dehydration in nisin H. Only in the small amount of the ninefold dehydrated species the Thr_2_ is dehydrated, in all other species we found a mix in the dehydration pattern, where Thr_2_ partially escape the dehydration. For example in the eightfold species we have a mix of dehydrated Thr_2_ or Ser_33_. In the nisin H F_1_I variant the Thr_2_ was in all species dehydrated, which suggests that, the phenylalanine at position one in nisin H is critical for the dehydratase NisB. Ser_29_ was never dehydrated in the found species.

**FIGURE 4:**
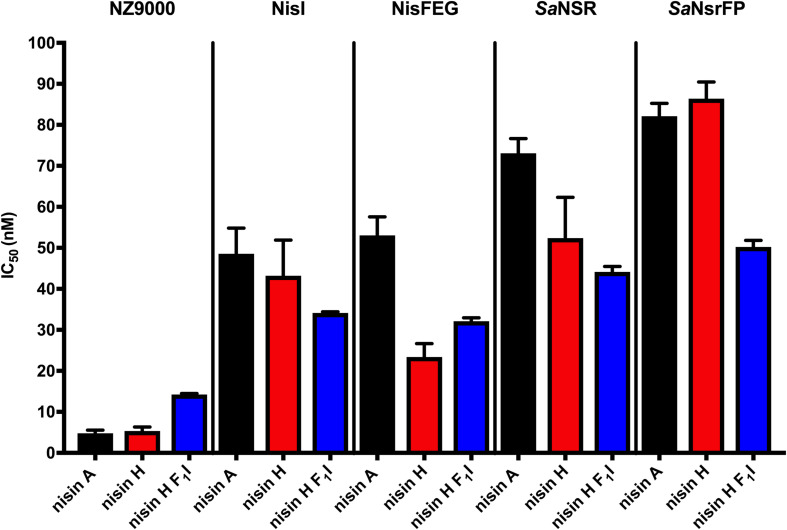
Growth inhibition assays in the presence of nisin A and the nisin H varants. The lantibiotic nisin A (black bars), the heterologous expressed nisin H (red bars) and the position 1 variant of nisin H F_1_I (blue bars) were used for growth inhibition (IC_50_) with the strains NZ9000Cm, NZ9000NisI, NZ9000NisFEG, NZ9000*Sa*NSR, and NZ9000*Sa*NsrFP. Values represent the average of at least five biological independent measurements and the errors report the standard deviation of the mean (SDM).

Lantibiotics are very potent and possess an antimicrobial activity in the nanomolar range (Gross and Morell, 1967; Rollema et al., 1995; Chan et al., 1996; Lu et al., 2010; Oppedijk et al., 2016). To verify this potential for nisin H and the nisin H F_1_I variant we used a standardized growth inhibition assay and first screened against the nisin sensitive *L. lactis* strain NZ9000Cm. Here, Cm stands for chloramphenicol resistance, which arises from the empty plasmid, which was transformed. In comparison to nisin A (IC_50_ value: 4.8 ± 0.7 nM), the heterologous expressed variant nisin H possessed a comparable IC_50_ value (5.3 ± 1.0 nM) (Figure 4 and Table 2). Both values are in line with previously determined IC_50_ values for the strain NZ9000Cm (Reiners et al., 2017). The effect of the nisin H F_1_I variant was more pronounced. For the NZ9000Cm sensitive strain we calculated an IC_50_ value of 14.2 ± 0.2 nM, approximately threefold lower than the wild-type nisin H (Figure 4 and Table 2).

**TABLE 2:** IC_50_ values (nM) for nisin A, nisin H, and nisin H F_1_I with the corresponding fold of resistance (FR) against the strains NZ9000Cm, NZ9000NisI, NZ9000NisFEG, NZ9000*Sa*NSR, and NZ9000*Sa*NsrFP.

**Variant**	**NZ9000Cm**	**NZ9000NisI**		**NZ9000NisFEG**		**NZ9000*Sa*NSR**		**NZ9000*Sa*NsrFP**	
		*IC*_50_	*FR*	*IC*_50_	*FR*	*IC*_50_	*FR*	*IC*_50_	*FR*
Nisin A	4.8 ± 0.7	48.6 ± 6.3	10.1 ± 2.0	53.0 ± 4.5	11.1 ± 1.9	73.0 ± 3.6	15.2 ± 2.5	82.1 ± 3.1	17.1 ± 2.7
Nisin H	5.3 ± 1.0	43.2 ± 8.7	8.1 ± 2.2	23.4 ± 3.3	4.4 ± 1.0	52.4 ± 9.9	9.8 ± 2.6	86.4 ± 4.1	16.2 ± 3.1
Nisin H F_1_I	14.2 ± 0.2	34.1 ± 0.3	2.4 ± 0.1	32.1 ± 0.8	2.3 ± 0.1	44.2 ± 1.3	3.1 ± 0.1	50.2 ± 1.6	3.5 ± 0.1

To test the effect of the nisin variants on the immunity proteins NisI (Alkhatib et al., 2014a) and NisFEG (Alkhatib et al., 2014b), as well as the resistance proteins *Sa*NSR (Khosa et al., 2016a) and *Sa*NsrFP (Reiners et al., 2017), we expressed each of them in a plasmid-based system in a *L. lactis* NZ9000 strain. We termed these strains NZ9000NisI, NZ9000NisFEG, NZ9000*Sa*NSR and NZ9000*Sa*NsrFP, respectively.

Nisin A displayed an IC_50_ value of 48.6 ± 6.3 nM against strain NZ9000NisI and 53.0 ± 4.5 nM against strain NZ9000NisFEG. For the resistance strains NZ9000*Sa*NSR and NZ9000*Sa*NsrFP nisin A displayed IC_50_ values of 73.0 ± 3.6 and 82.1 ± 3.1 nM, respectively (Figure 4 and Table 2). By comparing theses values, we calculated the fold of immunity/resistance to 10.1 ± 2.0 for NZ9000NisI, 11.1 ± 1.9 for NZ9000NisFEG, 15.2 ± 2.5 for NZ9000*Sa*NSR and 17.1 ± 2.7 for NZ9000*Sa*NsrFP (Table 2). After the first screen against the sensitive strain NZ9000Cm, nisin H and its variant were used to screen against the strains expressing the immunity or resistance proteins.

Nisin H revealed an IC_50_ value of 43.2 ± 8.7 nM against the NZ9000NisI strain, similar to nisin A, and a fold of immunity of 8.1 ± 2.2. Against the NZ9000NisFEG strain we determined an IC_50_ value of 23.4 ± 3.3 nM for nisin H which displayed a fold of resistance of 4.4 ± 1.0. Against the NZ9000*Sa*NSR strain we obtained an IC_50_ value of 52.4 ± 9.9 nM, resulting in a fold of resistance of 9.8 ± 2.6. Nisin H showed an IC_50_ value of 86.4 ± 4.0 nM for the NZ9000*Sa*NsrFP strain, resulting in a fold of resistance of 16.2 ± 3.1 (Figure 4 and Table 2). NZ9000*Sa*NsrFP showed the highest fold of resistance for nisin H [in-line with a previous report (Reiners et al., 2017)]. Intriguingly, strain NZ9000NisFEG showed a reduced immunity and consequently we propose that nisin H is not recognized as efficiently as nisin A. Even NZ9000*Sa*NSR had a reduced resistance. That could be due to the exchange of His_31_ against lysine in the C-terminal part of nisin H (Figure 1).

Surprisingly, all strains displayed a reducing resistance/immunity effect for the nisin H F_1_I variant in comparison to nisin A and also, with exception of NZ9000FEG for nisin H. Against the NZ9000NisI strain we determined an IC_50_ value of 34.1 ± 0.3 nM for the nisin H F_1_I variant, with a fold of resistance 2.4 ± 0.1, which is nearly threefold lower than for nisin A. We obtained an IC_50_ value of 32.1 ± 0.8 nM against the ABC transporter NZ9000NisFEG, with a fold of resistance 2.3 ± 0.1 (Figure 4 and Table 2). Nisin H F_1_I showed for the resistance strain NZ9000*Sa*NSR and NZ9000*Sa*NsrFP an IC_50_ value of 44.2 ± 1.3 and 50.2 ± 1.6 nM, respectively. The calculated folds of resistance were 3.1 ± 0.1 and 3.5 ± 0.1, respectively (Figure 4 and Table 2) and both are fivefold less than the observed fold of resistances for nisin A.

**FIGURE 5:**
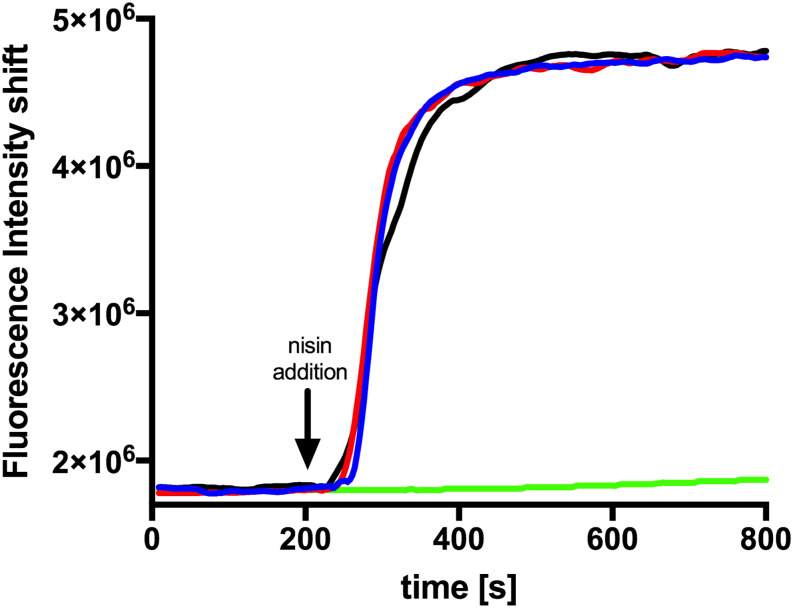
Nisin mediated pore formation, visualized with the SYTOX green assay. The NZ9000Cm strain incubated with the SYTOX dye. After a stable baseline (∼200 s), one of the nisin variants (100 nM) was added (indicated with an arrow). The fluorescence signal was measured using a fluorolog (Horiba III). The rapid increase in fluorescence indicates pore formation. The black line represents the addition of nisin A, the red line nisin H, and the blue line nisin H F_1_I. As a control we added buffer shown as green line.

Since a similar activity for nisin H and nisin A was observed it became obvious that both exhibit the same mode of action. In the case of nisin A this combines growth inhibition with pore formation in the membrane with subsequent cell death. To test this we performed a SYTOX assay previously used for nisin A (Reiners et al., 2017). Here the SYTOX dye was added to *L. lactis* cells and displayed an increased fluorescence signal upon binding of DNA which is released from the cell upon pore formation (Roth et al., 1997). We use 100 nM of nisin A, nisin H and nisin H F_1_I variant respectively and observed an almost instant fluorescence increase similar to the signal increase observed for nisin A (Figure 5). This shows that nisin H as well as its F_1_I variant form pores in the membrane of *L. lactis* cell.

The nisin variants were further tested for antibacterial capabilities against *B. subtilis* and different pathogenic strains from *S. aureus*, *E. faecium*, and *E. faecalis* using the microdilution method, according to the recommendations of CLSI (2012). Here we found that nisin H and the F_1_I variant performed almost identically or in most cases even better than the natural nisin A. Especially against the MSSA and MRSA strains, nisin H had significant lower MIC values of 0.19 and 0.78 μM, in comparison to 0.78 and 6.25 μM for nisin A, respectively (Table 3). Also, against *E. faecium* ATCC 35667, *B. subtilis* 168 as well as *E. faecium* ATCC 700221 (VRE), nisin H showed more potency with about two to eightfold lower MIC values than nisin A [0.39, 0.1, and 0.39 μM compared to 1.56, 0.78, and 0.78 μM for nisin A, respectively (Table 3)]. While the nisin H F_1_I variant and nisin A had similar MIC values against both MSSA and MRSA strains, the nisin H F_1_I variant only performed better than nisin A or nisin H against *E. faecalis* ATCC 51299 (VRE) with a MIC value of 0.78 μM compared to 1.56 μM for nisin A and nisin H. Against *E. faecium* ATCC 35667 and *B. subtilis* 168 nisin H F_1_I was less efficient than nisin H, but still better than nisin A (Table 3).

**TABLE 3:** MIC values for nisin A, nisin H, and nisin H F_1_I against different pathogenic strains.

**Organisms**	**Minimum inhibitory concentration (μM)**		
	**Nisin A**	**Nisin H**	**Nisin H F_1_I**
*Staphylococcus aureus* ATCC 29213 (MSSA)	0.78	0.19	0.78
*Staphylococcus aureus* ATCC 700699 (MRSA)	6.25	0.78	6.25
*Enterococcus faecalis* ATCC 29212	1.56	1.56	1.56
*Enterococcus faecium* ATCC 35667	1.56	0.39	0.78
*Bacillus subtilis* 168	0.78	0.1	0.39
*Enterococcus faecalis* ATCC 51299 (VRE)	1.56	1.56	0.78
*Enterococcus faecium* ATCC 700221 (VRE)	0.78	0.39	0.78

## Discussion

We focused in this study on the natural variant nisin H and the nisin H F_1_I mutant. Nisin H was discovered from the gut-derived strain *S. hyointestinalis* DPC6484 in 2015 by O’Connor et al. (2015). Here, we showed the heterologous expression of nisin H and the F_1_I variant with the NICE-system in *L. lactis* (Eichenbaum et al., 1998; Mierau and Kleerebezem, 2005; Rink et al., 2005; Zhou et al., 2006; Lagedroste et al., 2019) and extended the characterization in terms of cleavage efficiency by the protease NisP and the antimicrobial activity against the immunity proteins NisFEG (Alkhatib et al., 2014b) and NisI (Alkhatib et al., 2014a), as well as the resistance proteins *Sa*NSR (Khosa et al., 2013; Khosa et al., 2016a,b) and *Sa*NsrFP (Khosa et al., 2016a; Reiners et al., 2017). We further tested for antibacterial capabilities against *B. subtilis* and different pathogenic strains from *S. aureus*, *E. faecium*, and *E. faecalis.*

Both lantibiotics, nisin H and the F_1_I variant were purified in high amounts and purity with 5.3 ± 0.6 mg/L of cell culture for nisin H and 4.9 ± 0.2 mg/L of cell culture for nisin H F_1_I variant, respectively (Figure 2B and Table 1). In comparison, the homologous expression of nisin H in *S. hyointestinalis* and nisin A in *L. lactis* NZ9700 results in a very low amount of 0.15 mg/L of cell culture and 0.50 mg/L of cell culture (O’Connor et al., 2015), respectively, which demonstrates the enormous potential of the NICE-system, for lantibiotic and even non-lantibiotic expression (Eichenbaum et al., 1998; Mierau and Kleerebezem, 2005; Rink et al., 2005; Zhou et al., 2006; Lagedroste et al., 2019).

An important step in the maturation of a lantibiotic is the cleavage of the leader peptide to become biological active. The cleavage efficiency of the natural substrate nisin A was determined with 94.6 ± 2.0%. For nisin H the cleaving efficiency was drastically reduced to 15.6 ± 1.4%. The first residue of nisin A is an isoleucine, while the corresponding residue in nisin H is phenylalanine and as demonstrated before (Lagedroste et al., 2019), aromatic residues prevent efficient cleavage likely by interfering with the S1′ binding pocket of NisP. With the nisin H F_1_I variant, the cleavage efficiency was restored to 91.8 ± 0.8% (Figures 1, 2C and Table 1). This indicated that the other point mutations naturally occurring in nisin H did not affect cleavage by NisP. With respect to other natural nisin variants, NisP cleavage could be a critical step. For example nisin O1 to O4 from *B. obeum* A2-162 (Hatziioanou et al., 2017) has a tyrosine or a threonine, respectively, at position one, which should also result in a low NisP cleavage efficiency. Natural variants such as nisin U (Wirawan et al., 2006), nisin J (O’Sullivan et al., 2019, 2020), nisin Q (Zendo et al., 2003), nisin Z (Mulders et al., 1991) and nisin F (de Kwaadsteniet et al., 2008) have an isoleucine and nisin U2 (Wirawan et al., 2006) and nisin P (Zhang et al., 2012; Wu et al., 2014) a valine at position one, which should result in high NisP cleavage efficiency.

**FIGURE 6:**
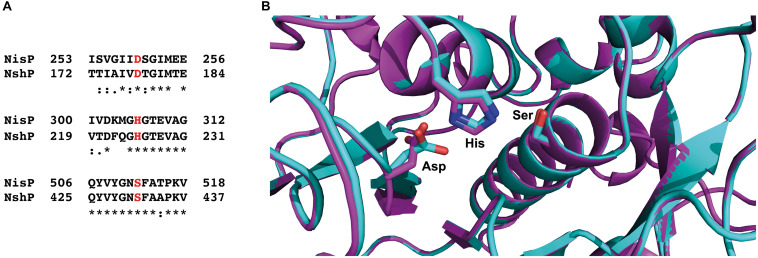
Sequence alignment of NisP from *Lactococcus lactis* and NshP from *Streptococcus hyointestinalis*. **(A)** The important residues from the active site (Asp_259_, His_306_, and Ser_512_ for NisP and Asp_178_, His_225_, and Ser_431_ for NshP) are marked in red, shown together with the close neighborhood residues. The sequence alignment was done with Clustal Omega (Madeira et al., 2019). **(B)** Zoom-in into the active sites of NshP (magenta) and NisP (cyan). The important residues for the catalytic activity (Asp_259_, His_306_, and Ser_512_ for NisP and Asp_178_, His_225_, and Ser_431_ for NshP) are shown in stick representation. Figure was generated using Pymol (2015). For a full alignment and a corresponding homology model, please see Supplementary Material.

Furthermore, we made a sequence alignment with Clustal Omega (Madeira et al., 2019) for the NshP (the natural protease for the nisin H cleavage) from *S. hyointestinalis* and NisP from *L. lactis* to see if there is any difference in the active site, which could be the reason for the reduced cleavage efficiency (Figure 6A and Supplementary Figure 1). Here the three important residues His_306_, Asp_259_, and Ser_512_ which build up the catalytic triad in NisP are conserved in NshP. We also calculated a homology model of NshP based on the known NisP structure (PDB code 4MZD) using Phyre2 (Kelley et al., 2015; Figure 6B and Supplementary Figure 2). Here, no significant differences are found within the overall fold as well as the active site which would explain the difference in the cleavage site. This is intriguing since the recognition site within the leader peptide differs between nisin A (sequence is ASPR) and nisin H (sequence is ASTR) (see Supplementary Figure 3). This suggests that the proteases NisP and NshP recognize their substrate by small difference in their active site.

Nevertheless, for an efficient cleavage, (methyl)-lanthionine rings have to be present. This even holds true in light of the presence of all (methyl)-lanthionine rings, which is generally assumed as the prerequisite for fast and efficient conversion of the pre-nisin to modified nisin (Plat et al., 2011; Lagedroste et al., 2017). To check the amount of dehydrations, necessary for (methyl)-lanthionine ring formation we applied MALDI-TOF analysis. The loss of water molecules within the peptide is directly visible in the reduced molecular weight, but not the (methyl)-lanthionine ring formation. Here we used 1-cyano-4 dimethylaminopyridinium tetrafluoroborate (CDAP) (Wu and Watson, 1998), which binds to free cysteine residues, indicating that these cysteines are not involved in a (methyl)-lanthionine ring. For both lantibiotics, nisin H and the nisin H F_1_I variant no CDAP coupling products were found, indicating that no (methyl)-lanthionine ring is lacking (Figure 3 and Table 1). Nisin H has 10 possible dehydration sites and is predicated to be ninefold dehydrated when expressed homologous (O’Connor et al., 2015). A minor species of nine dehydrations was found, but the dominant species was eightfold dehydrated. The dehydration pattern of the F_1_I variant is changed in comparison to nisin H. Here we determined a dominant ninefold species (Figure 3 and Table 1). This provides a hint, that position two of wild-type nisin H might not have been previously dehydrated, due to steric hindrance of the phenylalanine. To validate which serine or threonine residues is dehydrated, we perform a tandem mass spectrometric analysis of nisin H and the F_1_I variant. Here we found that the Thr_2_ partially escape the dehydration in nisin H. In the nisin H F_1_I variant the Thr_2_ was in all species dehydrated, which gives a hint that, the phenylalanine at position one in nisin H is critical for the dehydratase NisB. This is in line with previous data from the I_1_F variant of nisin A, where the dominant species was sevenfold dehydrated and not eightfold as wild-type nisin A (Lagedroste et al., 2019). It has also been reported for the natural nisin Z (Mulders et al., 1991), that the I_1_W mutation showed a partial inhibition of dehydration of the Thr_2_ (Breukink et al., 1998), which could also be the case for nisin H with the aromatic phenylalanine at position one, resulting in eight dehydrations. A dehydration of position Ser_29_ normally goes in line with the lack of ring E (Lubelski et al., 2009), which drastically reduces the antimicrobial activity of nisin A against the sensitive NZ9000Cm strain (Alkhatib et al., 2014b; Khosa et al., 2016a; Reiners et al., 2017). Since the activity was high for nisin H and the F_1_I variant, we expected that Ser_29_ was not dehydrated and tandem mass spectrometric analysis supported this.

Nisin H showed nearly the same activity as nisin A against the sensitive NZ9000Cm strain but the nisin H F_1_I variant is roughly threefold less active. For the immunity protein NisI, it was revealed that nisin H has an identical activity like nisin A within experimental error. However, the nisin H F_1_I variant exhibited a lower IC_50_ value of 34.1 ± 0.3 nM and due to the weaker wild-type activity more than a threefold lower fold of resistance (2.4 ± 0.1 compared to 8.1 ± 2.2) (Figure 4 and Table 2). NisI recognizes the N-terminus of nisin (Wiedemann et al., 2001) and the lower IC_50_ could be due to the fact that Thr_2_ is dehydrated in the nisin H F_1_I variant in contrast to nisin H. An additional change is the leucine at position 6 against methionine in nisin H and the nisin H F_1_I variant, which could be responsible for the better recognition by NisI.

The immunity protein NisFEG, in comparison to nisin A, showed a strong reduction in immunity in the presence of nisin H and the nisin H F_1_I variant. NisFEG recognizes the C-terminus of nisin (Alkhatib et al., 2014b), which indicates that the point mutations of nisin H affect NisFEG. So, we suppose that nisin H and the nisin H F_1_I variant are not recognized and subsequently transported out of the membrane like nisin A.

The resistance protein *Sa*NSR also recognizes the C-terminus of nisin (Khosa et al., 2016a), and cleaves nisin between the positions 28 and 29. Other studies showed that mutations in this area of the nisin molecule, e.g., S_29_P or C_28_P strongly reduce the efficiency of *Sa*NSR (Field et al., 2019; Zaschke-Kriesche et al., 2019a). We assume that the exchange of His_31_ against lysine in nisin H and the nisin H F_1_I variant (Figure 1) has the same effect on *Sa*NSR thereby lowering the resistance efficiency to an IC_50_ value of 52.4 ± 2.6 nM for nisin H and 44.2 ± 1.3 nM for the nisin H F_1_I variant, respectively (Figure 4 and Table 2).

For the resistance protein *Sa*NsrFP, we observed an activity for nisin H identical to nisin A. *Sa*NsrFP recognizes the N-terminus of nisin (Reiners et al., 2017), which is affected due to the different dehydration pattern in wild-type nisin H in comparison to the nisin H F_1_I variant. The nisin H F_1_I variant showed a lower IC_50_ value of 50.2 ± 1.6 nM, compared to 86.4 ± 4.1 nM for nisin H. This effect is even more pronounced when comparing the fold of resistances of 16.2 ± 3.1 for nisin H to 3.5 ± 0.1 for the nisin H F_1_I variant, respectively (Figure 4 and Table 2).

This study demonstrated again that only a complete characterization of a lantibiotic reveals the full antimicrobial potential. Based on the IC_50_ value of the sensitive NZ9000Cm strain the F_1_I variant might be classified as weakly antimicrobial active, but with respect to the immunity and resistance proteins, it becomes more interesting, due to its high activity even against the immunity proteins NisI and NisFEG from *L. lactis* and the nisin resistance proteins *Sa*NSR and *Sa*NsrFP from *S. agalactiae* COH1. Against the tested pathogenic bacteria, we found that nisin H and the nisin H F_1_I variant performed almost identically or in the most cases even better than the natural nisin A. Nisin H displayed high antimicrobial potential against both methicillin-resistant and –susceptible the *S. aureus* strains, both vancomycin-resistant and -susceptible, *E. faecium* strains, as well as *B. subtilis.*

## Data Availability Statement

The raw data supporting the conclusions of this article will be made available by the authors, without undue reservation.

## Author Contributions

LS and SS conceived and directed this study. JR and ML conducted the expression, purification, MS-analysis, and the growth inhibition experiments. JG performed the SYTOX experiments. GP and KS performed the tandem mass spectrometric analysis. EA and RK performed the MIC experiments. JR, ML, SS, and LS wrote the manuscript. All authors read and approved the manuscript.

## Conflict of Interest

The authors declare that the research was conducted in the absence of any commercial or financial relationships that could be construed as a potential conflict of interest.

## References

[B1] Abts A. Montalban-Lopez M. Kuipers O. P. Smits S. H. Schmitt L. (2013). NisC binds the FxLx motif of the nisin leader peptide. *Biochemistry* 52 5387–5395. 10.1021/bi4008116 23869662

[B2] Alkhatib Z. Lagedroste M. Fey I. Kleinschrodt D. Abts A. Smits S. H. (2014a). Lantibiotic immunity: inhibition of nisin mediated pore formation by NisI. *PLoS One* 9:e102246. 10.1371/journal.pone.0102246 25014359 PMC4094520

[B3] Alkhatib Z. Lagedroste M. Zaschke J. Wagner M. Abts A. Fey I. (2014b). The C-terminus of nisin is important for the ABC transporter NisFEG to confer immunity in *Lactococcus lactis*. *Microbiologyopen* 3 752–763. 10.1002/mbo3.205 25176038 PMC4234265

[B4] Arnison P. G. Bibb M. J. Bierbaum G. Bowers A. A. Bugni T. S. Bulaj G. (2013). Ribosomally synthesized and post-translationally modified peptide natural products: overview and recommendations for a universal nomenclature. *Nat. Prod. Rep.* 30 108–160. 23165928 10.1039/c2np20085f PMC3954855

[B5] Breukink E. Van Kraaij C. Van Dalen A. Demel R. A. Siezen R. J. De Kruijff B. (1998). The orientation of nisin in membranes. *Biochemistry* 37 8153–8162. 10.1021/bi972797l 9609711

[B6] Brunati C. Thomsen T. T. Gaspari E. Maffioli S. Sosio M. Jabes D. (2018). Expanding the potential of NAI-107 for treating serious ESKAPE pathogens: synergistic combinations against Gram-negatives and bactericidal activity against non-dividing cells. *J. Antimicrob. Chemother.* 73 414–424. 10.1093/jac/dkx395 29092042 PMC5890740

[B7] Chan W. C. Leyland M. Clark J. Dodd H. M. Lian L. Y. Gasson M. J. (1996). Structure-activity relationships in the peptide antibiotic nisin: antibacterial activity of fragments of nisin. *FEBS Lett.* 390 129–132. 10.1016/0014-5793(96)00638-2 8706842

[B8] Clinical and Laboratory Standards Institute (2012). *Methods for Dilution Antimicrobial Susceptibility Tests for Bacteria that Grow Aerobically; Approved Standard*, 9th Edn Wayne PA: Clinical and Laboratory Standards Institute.

[B9] Crowther G. S. Baines S. D. Todhunter S. L. Freeman J. Chilton C. H. Wilcox M. H. (2013). Evaluation of NVB302 versus vancomycin activity in an in vitro human gut model of Clostridium difficile infection. *J. Antimicrob. Chemother.* 68 168–176. 10.1093/jac/dks359 22966180

[B10] Dawson M. J. Scott R. W. (2012). New horizons for host defense peptides and lantibiotics. *Curr. Opin. Pharmacol.* 12 545–550. 10.1016/j.coph.2012.06.006 22776251 PMC3466353

[B11] de Kwaadsteniet M. Ten Doeschate K. Dicks L. M. (2008). Characterization of the structural gene encoding nisin F, a new lantibiotic produced by a *Lactococcus lactis* subsp. lactis isolate from freshwater catfish (*Clarias gariepinus*). *Appl. Environ. Microbiol.* 74 547–549. 10.1128/aem.01862-07 18039827 PMC2223265

[B12] Delves-Broughton J. Blackburn P. Evans R. J. Hugenholtz J. (1996). Applications of the bacteriocin, nisin. *Antonie Van Leeuwenhoek* 69 193–202. 10.1007/bf00399424 8775979

[B13] Dischinger J. Basi Chipalu S. Bierbaum G. (2014). Lantibiotics: promising candidates for future applications in health care. *Int. J. Med. Microbiol.* 304 51–62. 10.1016/j.ijmm.2013.09.003 24210177

[B14] Eichenbaum Z. Federle M. J. Marra D. De Vos W. M. Kuipers O. P. Kleerebezem M. (1998). Use of the lactococcal nisA promoter to regulate gene expression in gram-positive bacteria: comparison of induction level and promoter strength. *Appl. Environ. Microbiol.* 64 2763–2769. 10.1128/aem.64.8.2763-2769.1998 9687428 PMC106770

[B15] Field D. Blake T. Mathur H. Pm O. C. Cotter P. D. Paul Ross R. (2019). Bioengineering nisin to overcome the nisin resistance protein. *Mol. Microbiol.* 111 717–731. 30537404 10.1111/mmi.14183

[B16] Gross E. Morell J. L. (1967). The presence of dehydroalanine in the antibiotic nisin and its relationship to activity. *J. Am. Chem. Soc.* 89 2791–2792. 10.1021/ja00987a084 6043807

[B17] Hasper H. E. De Kruijff B. Breukink E. (2004). Assembly and stability of nisin-lipid II pores. *Biochemistry* 43 11567–11575. 10.1021/bi049476b 15350143

[B18] Hatziioanou D. Gherghisan-Filip C. Saalbach G. Horn N. Wegmann U. Duncan S. H. (2017). Discovery of a novel lantibiotic nisin O from Blautia obeum A2-162, isolated from the human gastrointestinal tract. *Microbiology* 163 1292–1305. 10.1099/mic.0.000515 28857034 PMC5882112

[B19] Holo H. Nes I. F. (1989). High-Frequency transformation, by electroporation, of *Lactococcus lactis* subsp. cremoris grown with glycine in osmotically stabilized media. *Appl. Environ. Microbiol.* 55 3119–3123. 10.1128/aem.55.12.3119-3123.1989 16348073 PMC203233

[B20] Hsu S. T. D. Breukink E. Tischenko E. Lutters M. A. G. De Kruijff B. Kaptein R. (2004). The nisin-lipid II complex reveals a pyrophosphate cage that provides a blueprint for novel antibiotics. *Nat. Struct. Mol. Biol.* 11 963–967. 10.1038/nsmb830 15361862

[B21] Jabes D. Brunati C. Candiani G. Riva S. Romano G. Donadio S. (2011). Efficacy of the new lantibiotic NAI-107 in experimental infections induced by multidrug-resistant Gram-positive pathogens. *Antimicrob. Agents Chemother.* 55 1671–1676. 10.1128/aac.01288-10 21220527 PMC3067139

[B22] Jensen P. R. Hammer K. (1993). Minimal requirements for exponential growth of *Lactococcus lactis*. *Appl. Environ. Microbiol.* 59 4363–4366. 10.1128/aem.59.12.4363-4366.1993 16349136 PMC195913

[B23] Kaletta C. Entian K. D. (1989). Nisin, a peptide antibiotic: cloning and sequencing of the nisA gene and posttranslational processing of its peptide product. *J. Bacteriol.* 171 1597–1601. 10.1128/jb.171.3.1597-1601.1989 2493449 PMC209786

[B24] Karakas Sen A. Narbad A. Horn N. Dodd H. M. Parr A. J. Colquhoun I. (1999). Post-translational modification of nisin. The involvement of NisB in the dehydration process. *Eur. J. Biochem.* 261 524–532. 10.1046/j.1432-1327.1999.00303.x 10215865

[B25] Kelley L. A. Mezulis S. Yates C. M. Wass M. N. Sternberg M. J. (2015). The Phyre2 web portal for protein modeling, prediction and analysis. *Nat. Protoc.* 10 845–858. 10.1038/nprot.2015.053 25950237 PMC5298202

[B26] Khosa S. Alkhatib Z. Smits S. H. (2013). NSR from Streptococcus agalactiae confers resistance against nisin and is encoded by a conserved nsr operon. *Biol. Chem.* 394 1543–1549. 10.1515/hsz-2013-0167 23893686

[B27] Khosa S. Frieg B. Mulnaes D. Kleinschrodt D. Hoeppner A. Gohlke H. (2016a). Structural basis of lantibiotic recognition by the nisin resistance protein from Streptococcus agalactiae. *Sci. Rep.* 6:18679. 10.1038/srep18679 PMC4698656 26727488

[B28] Khosa S. Lagedroste M. Smits S. H. (2016b). Protein defense systems against the lantibiotic nisin: function of the immunity protein NisI and the resistance protein NSR. *Front. Microbiol.* 7:504. 10.3389/fmicb.2016.00504 27148193 PMC4828448

[B29] Klaenhammer T. R. (1993). Genetics of bacteriocins produced by lactic acid bacteria. *FEMS Microbiol. Rev.* 12 39–85. 10.1016/0168-6445(93)90057-g 8398217

[B30] Koponen O. Tolonen M. Qiao M. Wahlstrom G. Helin J. Saris P. E. J. (2002). NisB is required for the dehydration and NisC for the lanthionine formation in the post-translational modification of nisin. *Microbiology* 148 3561–3568. 10.1099/00221287-148-11-3561 12427947

[B31] Kuipers O. P. De Ruyter P. G. Kleerebezem M. De Vos W. M. (1997). Controlled overproduction of proteins by lactic acid bacteria. *Trends Biotechnol.* 15 135–140. 10.1016/s0167-7799(97)01029-9 9131833

[B32] Lagedroste M. Reiners J. Smits S. H. J. Schmitt L. (2019). Systematic characterization of position one variants within the lantibiotic nisin. *Sci. Rep.* 9:935. 10.1038/s41598-018-37532-4 PMC6353901 30700815

[B33] Lagedroste M. Smits S. H. J. Schmitt L. (2017). Substrate specificity of the secreted nisin leader peptidase NisP. *Biochemistry* 56 4005–4014. 10.1021/acs.biochem.7b00524 28675292

[B34] Li B. van der Donk W. A. (2007). Identification of essential catalytic residues of the cyclase NisC involved in the biosynthesis of nisin. *J. Biol. Chem.* 282 21169–21175. 10.1074/jbc.m701802200 17513866

[B35] Li B. Yu J. P. Brunzelle J. S. Moll G. N. Van Der Donk W. A. Nair S. K. (2006). Structure and mechanism of the lantibiotic cyclase involved in nisin biosynthesis. *Science* 311 1464–1467. 10.1126/science.1121422 16527981

[B36] Lu Y. Jiang L. Chen M. Huan L. Zhong J. (2010). [Improving heat and pH stability of nisin by site-directed mutagenesis]. *Wei Sheng Wu Xue Bao* 50 1481–1487. 21268893

[B37] Lubelski J. Khusainov R. Kuipers O. P. (2009). Directionality and coordination of dehydration and ring formation during biosynthesis of the lantibiotic nisin. *J. Biol. Chem.* 284 25962–25972. 10.1074/jbc.m109.026690 19620240 PMC2757997

[B38] Madeira F. Park Y. M. Lee J. Buso N. Gur T. Madhusoodanan N. (2019). The EMBL-EBI search and sequence analysis tools APIs in 2019. *Nucleic Acids Res.* 47 W636–W641. 30976793 10.1093/nar/gkz268 PMC6602479

[B39] Medeiros-Silva J. Jekhmane S. Paioni A. L. Gawarecka K. Baldus M. Swiezewska E. (2018). High-resolution NMR studies of antibiotics in cellular membranes. *Nat. Commun.* 9:3963. 10.1038/s41467-018-06314-x PMC6160437 30262913

[B40] Mierau I. Kleerebezem M. (2005). 10 years of the nisin-controlled gene expression system (NICE) in *Lactococcus lactis*. *Appl. Microbiol. Biotechnol.* 68 705–717. 10.1007/s00253-005-0107-6 16088349

[B41] Mota-Meira M. Lapointe G. Lacroix C. Lavoie M. C. (2000). MICs of mutacin B-Ny266, nisin A, vancomycin, and oxacillin against bacterial pathogens. *Antimicrob. Agents Chemother.* 44 24–29. 10.1128/aac.44.1.24-29.2000 10602718 PMC89623

[B42] Mulders J. W. Boerrigter I. J. Rollema H. S. Siezen R. J. De Vos W. M. (1991). Identification and characterization of the lantibiotic nisin Z, a natural nisin variant. *Eur. J. Biochem.* 201 581–584. 10.1111/j.1432-1033.1991.tb16317.x 1935953

[B43] O’Sullivan J. N. O’Connor P. M. Rea M. C. O’Sullivan O. Walsh C. J. Healy B. (2020). Nisin J, a novel natural nisin variant, is produced by *Staphylococcus capitis* Sourced from the human skin microbiota. *J. Bacteriol* 202. 10.1128/JB.00639-19 31740495 PMC6964739

[B44] O’Connor P. M. O’Shea E. F. Guinane C. M. O’Sullivan O. Cotter P. D. Ross R. P. (2015). Nisin H is a new nisin variant produced by the gut-derived strain *Streptococcus hyointestinalis* DPC6484. *Appl. Environ. Microbiol.* 81 3953–3960. 10.1128/aem.00212-15 25841003 PMC4524162

[B45] Okeley N. M. Paul M. Stasser J. P. Blackburn N. Van Der Donk W. A. (2003). SpaC and NisC, the cyclases involved in subtilin and nisin biosynthesis, are zinc proteins. *Biochemistry* 42 13613–13624. 10.1021/bi0354942 14622008

[B46] Ongey E. L. Yassi H. Pflugmacher S. Neubauer P. (2017). Pharmacological and pharmacokinetic properties of lanthipeptides undergoing clinical studies. *Biotechnol. Lett.* 39 473–482. 10.1007/s10529-016-2279-9 28044226

[B47] Oppedijk S. F. Martin N. I. Breukink E. (2016). Hit ‘em where it hurts: the growing and structurally diverse family of peptides that target lipid-II. *Biochim. Biophys. Acta* 1858 947–957. 10.1016/j.bbamem.2015.10.024 26523408

[B48] Ortega M. A. Hao Y. Zhang Q. Walker M. C. Van Der Donk W. A. Nair S. K. (2015). Structure and mechanism of the tRNA-dependent lantibiotic dehydratase NisB. *Nature* 517 509–512. 10.1038/nature13888 25363770 PMC4430201

[B49] O’Sullivan J. N. Rea M. C. O’Connor P. M. Hill C. Ross R. P. (2019). Human skin microbiota is a rich source of bacteriocin-producing staphylococci that kill human pathogens. *FEMS Microbiol. Ecol.* 95:fiy241. 10.1093/femsec/fiy241 PMC6340406 30590567

[B50] Plat A. Kluskens L. D. Kuipers A. Rink R. Moll G. N. (2011). Requirements of the engineered leader peptide of nisin for inducing modification, export, and cleavage. *Appl. Environ. Microbiol.* 77 604–611. 10.1128/aem.01503-10 21097596 PMC3020565

[B51] Pymol (2015). *The PyMOL Molecular Graphics System, Version 2.0.* New York, NY: Schrödinger.

[B52] Reiners J. Lagedroste M. Ehlen K. Leusch S. Zaschke-Kriesche J. Smits S. H. J. (2017). The N-terminal region of nisin is important for the BceAB-Type ABC Transporter NsrFP from *Streptococcus agalactiae* COH1. *Front. Microbiol.* 8:1643. 10.3389/fmicb.2017.01643 28912758 PMC5583591

[B53] Repka L. M. Chekan J. R. Nair S. K. Van Der Donk W. A. (2017). Mechanistic understanding of lanthipeptide biosynthetic enzymes. *Chem. Rev.* 117 5457–5520. 10.1021/acs.chemrev.6b00591 28135077 PMC5408752

[B54] Rink R. Kuipers A. De Boef E. Leenhouts K. J. Driessen A. J. Moll G. N. (2005). Lantibiotic structures as guidelines for the design of peptides that can be modified by lantibiotic enzymes. *Biochemistry* 44 8873–8882. 10.1021/bi050081h 15952794

[B55] Rogers L. A. (1928). The inhibiting effect of streptococcus lactis on Lactobacillus Bulgaricus. *J. Bacteriol.* 16 321–325. 10.1128/jb.16.5.321-325.1928 16559344 PMC375033

[B56] Rogers L. A. Whittier E. O. (1928). Limiting factors in the lactic fermentation. *J. Bacteriol.* 16 211–229. 10.1128/jb.16.4.211-229.1928 16559334 PMC375023

[B57] Rollema H. S. Kuipers O. P. Both P. De Vos W. M. Siezen R. J. (1995). Improvement of solubility and stability of the antimicrobial peptide nisin by protein engineering. *Appl. Environ. Microbiol.* 61 2873–2878. 10.1128/aem.61.8.2873-2878.1995 7487019 PMC167563

[B58] Roth B. L. Poot M. Yue S. T. Millard P. J. (1997). Bacterial viability and antibiotic susceptibility testing with SYTOX green nucleic acid stain. *Appl. Environ. Microbiol.* 63 2421–2431. 10.1128/aem.63.6.2421-2431.1997 9172364 PMC168536

[B59] Sahl H. G. Bierbaum G. (1998). Lantibiotics: biosynthesis and biological activities of uniquely modified peptides from gram-positive bacteria. *Annu. Rev. Microbiol.* 52 41–79. 10.1146/annurev.micro.52.1.41 9891793

[B60] Sandiford S. K. (2019). Current developments in lantibiotic discovery for treating Clostridium difficile infection. *Expert Opin. Drug. Discov.* 14 71–79. 10.1080/17460441.2019.1549032 30479173

[B61] Terzaghi B. E. Sandine W. E. (1975). Improved medium for lactic Streptococci and Their Bacteriophages. *Appl. Microbiol.* 29 807–813. 10.1128/aem.29.6.807-813.1975 16350018 PMC187084

[B62] van Heel A. J. De Jong A. Song C. Viel J. H. Kok J. Kuipers O. P. (2018). BAGEL4: a user-friendly web server to thoroughly mine RiPPs and bacteriocins. *Nucleic Acids Res.* 46 W278–W281. 29788290 10.1093/nar/gky383 PMC6030817

[B63] van Heusden H. E. De Kruijff B. Breukink E. (2002). Lipid II induces a transmembrane orientation of the pore-forming peptide lantibiotic nisin. *Biochemistry* 41 12171–12178. 10.1021/bi026090x 12356318

[B64] Wiedemann I. Benz R. Sahl H. G. (2004). Lipid II-mediated pore formation by the peptide antibiotic nisin: a black lipid membrane study. *J. Bacteriol.* 186 3259–3261. 10.1128/jb.186.10.3259-3261.2004 15126490 PMC400633

[B65] Wiedemann I. Breukink E. Van Kraaij C. Kuipers O. P. Bierbaum G. De Kruijff B. (2001). Specific binding of nisin to the peptidoglycan precursor lipid II combines pore formation and inhibition of cell wall biosynthesis for potent antibiotic activity. *J. Biol. Chem.* 276 1772–1779. 10.1074/jbc.m006770200 11038353

[B66] Wirawan R. E. Klesse N. A. Jack R. W. Tagg J. R. (2006). Molecular and genetic characterization of a novel nisin variant produced by *Streptococcus uberis*. *Appl. Environ. Microbiol.* 72 1148–1156. 10.1128/aem.72.2.1148-1156.2006 16461661 PMC1392965

[B67] Wu J. Watson J. T. (1998). Optimization of the cleavage reaction for cyanylated cysteinyl proteins for efficient and simplified mass mapping. *Anal. Biochem.* 258 268–276. 10.1006/abio.1998.2596 9570840

[B68] Wu Z. Wang W. Tang M. Shao J. Dai C. Zhang W. (2014). Comparative genomic analysis shows that *Streptococcus suis* meningitis isolate SC070731 contains a unique 105K genomic island. *Gene* 535 156–164. 10.1016/j.gene.2013.11.044 24316490

[B69] Zaschke-Kriesche J. Behrmann L. V. Reiners J. Lagedroste M. Groner Y. Kalscheuer R. (2019a). Bypassing lantibiotic resistance by an effective nisin derivative. *Bioorg. Med. Chem.* 27 3454–3462. 10.1016/j.bmc.2019.06.031 31253534

[B70] Zaschke-Kriesche J. Reiners J. Lagedroste M. Smits S. H. J. (2019b). Influence of nisin hinge-region variants on lantibiotic immunity and resistance proteins. *Bioorg. Med. Chem.* 27 3947–3953. 10.1016/j.bmc.2019.07.014 31331652

[B71] Zendo T. Fukao M. Ueda K. Higuchi T. Nakayama J. Sonomoto K. (2003). Identification of the lantibiotic nisin Q, a new natural nisin variant produced by *Lactococcus lactis* 61-14 isolated from a river in Japan. *Biosci. Biotechnol. Biochem.* 67 1616–1619. 10.1271/bbb.67.1616 12913315

[B72] Zhang Q. Yu Y. Velasquez J. E. Van Der Donk W. A. (2012). Evolution of lanthipeptide synthetases. *Proc. Natl. Acad. Sci. U.S.A.* 109 18361–18366. 10.1073/pnas.1210393109 23071302 PMC3494888

[B73] Zhou L. Van Heel A. J. Kuipers O. P. (2015). The length of a lantibiotic hinge region has profound influence on antimicrobial activity and host specificity. *Front. Microbiol* 6:11. 10.3389/fmicb.2015.00011 25688235 PMC4310329

[B74] Zhou X. X. Li W. F. Ma G. X. Pan Y. J. (2006). The nisin-controlled gene expression system: construction, application and improvements. *Biotechnol. Adv.* 24 285–295. 10.1016/j.biotechadv.2005.11.001 16380225

