# Supplementary material for: Insights in the Antimicrobial Potential of the Natural Nisin Variant Nisin H
Source: Front Microbiol. 2020 Oct 20;11:573614. doi: 10.3389/fmicb.2020.573614 (PMC7606277; doi:10.3389/fmicb.2020.573614)
Supplement: Supplementary file 1 [file Data_Sheet_1.PDF]

Supplementary Material

Insights in the antimicrobial potential of the natural nisin variant nisin H

Jens Reiners[1,2]*, Marcel Lagedroste[1]*, Julia Gottstein[1], Emmanuel T. Adeniyi[3], Rainer Kalscheuer[3], Gereon Poschmann[4], Kai Stühler[4,5], Sander H.J. Smits[1,2 #] and Lutz Schmitt[1 #]

[1]Institute of Biochemistry, Heinrich-Heine-University Düsseldorf, Universitaetsstrasse 1, 40225, Düsseldorf, Germany.

[2]Center for Structural Studies, Heinrich-Heine-University Düsseldorf, Universitaetsstrasse 1, 40225, Düsseldorf, Germany.

[3]Institute of Pharmaceutical Biology and Biotechnology, Heinrich-Heine-University Düsseldorf, Universitaetsstrasse 1, 40225, Düsseldorf, Germany.

[4]Institute for Molecular Medicine, Medical Faculty, Heinrich-Heine-University Düsseldorf, 40225 Düsseldorf, Germany

[5]Molecular Proteomics Laboratory, BMFZ, Heinrich-Heine-University-Düsseldorf, 40225 Düsseldorf, Germany

*Contributed equally

#Address correspondence to Lutz Schmitt: lutz.schmitt@hhu.de or Sander Smits sander.smits@hhu.de

Key words: lantibiotics, nisin, nisin H, MS analysis, antimicrobial activity

```
NisP      ----MKKILGFLFIVCSLGLSATVHGETTNSQQLLSNNINTELINHNSNAILSSTEGSTT      56
NshP      MGNNVKKFLCLLCMFFTFSITPIVYGEDIS------------------------------      30
              :**:* :* :. ::.::  *:**  .

NisP      DSINLGAQSPAVKSTTRTELDVTGAAKTLLQTSAVQKEMKVSLQETQVSSEF--SKRDSV      114
NshP      ----------------------------------------LSVDKNEVTTQVSVSSSDEY      50
                                                  :*:::.:*:::.  *. *.

NisP      TNKEAVPVSKDELLEQSEVVVSTSSIQKNKILDNK--------KNRANFVTSSPLIKEKP      166
NshP      QSGDATPQNQYDVTKSSDSYD--NHVGSQSYIDNSNKSDTAEDTINSSYVEDSSIVSEQE      108
           . :*.* .: :: :.*:     . : .:. :**.        . .:.:* .* ::.*:

NisP      SNSKDASGVIDNSASPLSYRKAKEVVSLRQPLKNQKVEAQPLLISNSSEKKASVYTNSHD      226
NshP      S------GE------------SKEVD-SQENFQSDQVQ----TKETTADGNTNNLSASHI      145
          *      *             :***   :: ::.::*:      ..::: ::.  : **

NisP      FWDYQWDMKYVTNNGESYALYQPSKKISVGIIDSGIMEEHPDLSNSLGNYFKNLVPKGGF      286
NshP      FWDKQWDIKNVTNNGESYELYQASKNTTIAIVDTGIMTEHPDLAFSLGDYLKNFVPEGGF      205
          *** ***:* ******** *** **: ::.*:*:*** *****: ***:*:**:**:***

NisP      DNEEPDETGNPSDIVDKMGHGTEVAGQITANGNILGVAPGITVNIYRVFGENLSKSEWVA      346
NshP      DGSEAYEQGNVADVTDFQGHGTEVAGQITANGNMLGVAPGITINIYRVFGESYAKPEWIA      265
          *..*  * ** :*:.*  ***************:********:********. :* **:*

NisP      RAIRRAADDGNKVINISAGQYLMISGSYDDGTNDYQEYLNYKSAINYATAKGSIVVAALG      406
NshP      EAVKQAADDGNRVITISSGQYLMITGSYEDGTNDYQDYLIYKEAVDYATSKGSIVVAALG      325
          .*:::******:**.**:******:***:*******:** **.*::***:**********

NisP      NDSLNIQDNQTMINFLKRFRSIKVPGKVVDAPSVFEDVIAVGGIDGYGNISDFSNIGADA      466
NshP      NEGLNIQDNQALVDYVSTYRNIKVPGVVVDAPSVFDNVVAVGGIDYYNNLSDFSNYSSAA      385
          *:.*******::::::. :*.***** ********::*:****** *.*:***** .: *

NisP      IYAPAGTTANFKKYGQDKFVSQGYYLKDWLFTTTNTGWYQYVYGNSFATPKVSGALALVV      526
NshP      IYAPAGTTANLVKYGEEAFTNQGFYLTDWIFTTSYTGWYQYVYGNSFAAPKVAGALALVA      445
          **********: ***:: *..**:**.**:***: *************:***:******.

NisP      DKYGIKNPNQLKRFLLMNSPEVNGNRVLNIVDLLNGKNKAFSLDTDKGQDDAINHKSMEN      586
NshP      DKYNITDVKQLKEYLISHSPEINSLKILNIVDLLSEDIKTVYTEII-------YKKA---      495
          ***.*.: :***.:*: :***:*. ::*******. . *:.  :          :*:

NisP      LKESRDTMKQEQDKEIQRNTNNNFSIKNDFHNISKEVISVDYNINQKMANNRNSRGAVSV      646
NshP      LIESRVEV-----------SLEVLNMSKKFNDVTPDNYIAKYNSKNSENNKINDRGVVSL      544
          * ***  :           : : :.:.:.*:::: :   ..** ::.  *: *.**.**:

NisP      RSQEILPVTGDGEDFL-PALGIVCISILGILKRKTKN-                            682
NshP      RSCETLPNTGANRDVTYEVLGLFLLAISTSWKKKMKTE                            582
          ** * ** ** ..*.   .**:. ::*    *:* *.
```

Figure S1: Sequence alignment of NispP from *Lactococcus lactis* and NshP from *Streptococcus hyointestinalis*. The important residues of the active site (Asp$_{259}$, His$_{306}$, and Ser$_{512}$ for NisP and Asp$_{178}$, His$_{225}$, and Ser$_{431}$ for NshP) are highlighted in red. The sequence alignment was done with Clustal Omega [1].

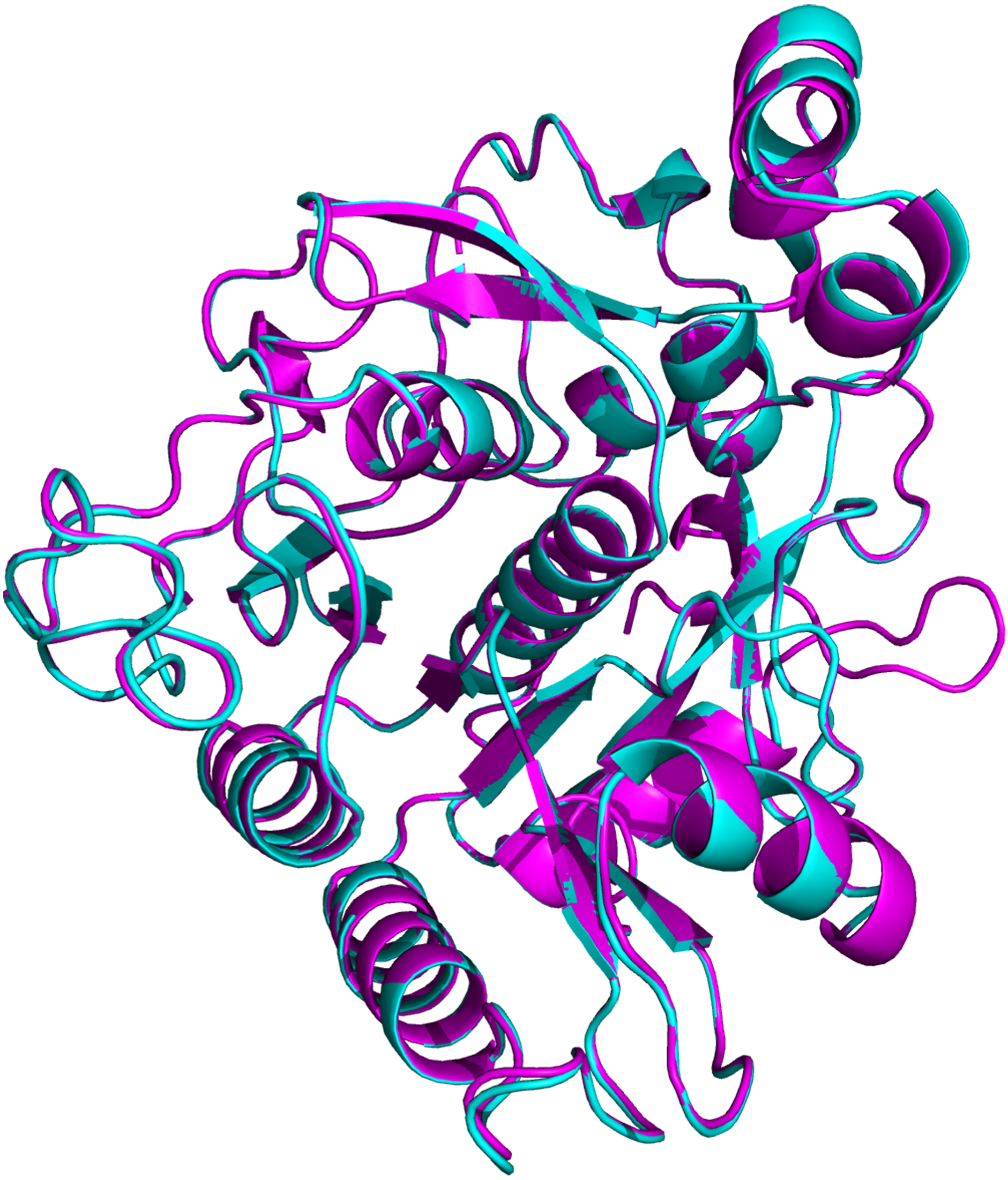

Figure S2: Homology model of NshP from *Streptococcus hyointestinalis*. A homology model of NshP was created using Phyre2 [2]. We used the sequence of the active protein without the self-cleaving part. The NshP model is shown in magenta and NisP (PDB code: 4MZD) in cyan. Figure was generated using PyMol [3].

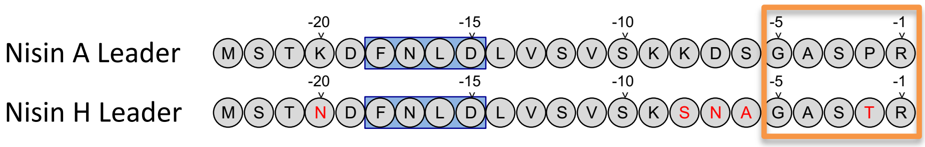

Nisin A Leader MSTKDFNLDLVSVSKKDSGASPR
Nisin H Leader MSTNDFNLDLVSVSKSNAGASTR

Figure S3: Sequence of nisin A and Nisin H leader sequence. Highlighted are the FNLD box (blue box) known to be important for the modification enzymes as well differences in sequence indicated by red letters. The cleavage site within the leader sequence of nisin A and nisin H is highlighted by an orange box.

1. Madeira, F., et al., *The EMBL-EBI search and sequence analysis tools APIs in 2019.* Nucleic Acids Res, 2019. **47**(W1): p. W636-W641.
2. Kelley, L.A., et al., *The Phyre2 web portal for protein modeling, prediction and analysis.* Nat Protoc, 2015. **10**(6): p. 845-58.
3. *The PyMOL Molecular Graphics System, Version 2.0 Schrödinger, LLC.*
